# The impact of the COVID-19 pandemic on neuropsychiatric and sleep disorders, and quality of life in individuals with neurodegenerative and demyelinating diseases: a systematic review and meta-analysis of observational studies

**DOI:** 10.1186/s12883-023-03176-9

**Published:** 2023-04-12

**Authors:** Marcos Paulo Braz de Oliveira, Ana Emilia Fonseca de Castro, Andressa Leticia Miri, Carla Rigo Lima, Brendon David Truax, Vanessa Suziane Probst, Suhaila Mahmoud Smaili

**Affiliations:** 1 Healthy Aging Research Laboratory, Department of Physical Therapy, Federal University of São Carlos, Washington Luis Highway, Km 235, São Carlos São Paulo, Brazil; 2 Physical Therapy Laboratory, Department of Physical Therapy, State University of Campinas, São Paulo, Campinas Brazil; 3 Neurofunctional Physical Therapy Research Group, Department of Physical Therapy, State University of Londrina, Londrina, Paraná Brazil; 4 Mechanisms of Spinal Manual Therapy Laboratory, Department of Physical Therapy, The University of Alabama at Birmingham, Birmingham, AL USA; 5 Department of Medicine, Division of Pulmonary, Allergy and Critical Care, The University of Alabama at Birmingham, Birmingham, AL USA

**Keywords:** Parkinson disease, Multiple sclerosis, Alzheimer disease, COVID-19, Systematic review, Meta-analysis

## Abstract

**Background:**

The coronavirus disease 2019 (COVID-19) pandemic has affected the mental health, sleep and quality of life, especially in individuals with chronic disease. Therefore, the purpose of this systematic review and meta-analysis was to investigate the impact of the COVID-19 pandemic on neuropsychiatric disorders (depression, anxiety, stress), sleep disorders (sleep quality, insomnia) and quality of life in individuals with Parkinson’s disease (PD), Multiple Sclerosis (MS) and Alzheimer's disease (AD) compared to healthy controls.

**Methods:**

Seven databases (Medline, Embase, ScienceDirect, Web of Science, The Cochrane Library, Scielo and Lilacs) were searched between March 2020 and December 2022. Observational studies (i.e., cross-sectional, case–control, cohort) were included. GRADE approach was used to assess the quality of evidence and strength of the recommendation. Effect size was calculated using standardized mean differences (SMD; random effects model). A customized Downs and Black checklist was used to assess the risk of bias.

**Results:**

Eighteen studies (PD = 7, MS = 11) were included. A total of 627 individuals with PD (healthy controls = 857) and 3923 individuals with MS (healthy controls = 2432) were analyzed. Twelve studies (PD = 4, MS = 8) were included in the meta-analysis. Individuals with PD had significantly elevated levels of depression (very low evidence, SMD = 0.40,* p* = 0.04) and stress (very low evidence, SMD = 0.60,* p* < 0.0001). There was no difference in anxiety (*p* = 0.08). Individuals with MS had significantly higher levels of depression (very low evidence, SMD = 0.73,* p* = 0.007) and stress (low evidence, SMD = 0.69,* p* = 0.03) and low quality of life (very low evidence, SMD = 0.77,* p* = 0.006). There was no difference in anxiety (*p* = 0.05) and sleep quality (*p* = 0.13). It was not possible to synthesize evidence in individuals with AD and sleep disorder (insomnia).

**Conclusion:**

In general, the COVID-19 pandemic negatively impacted individuals with PD and MS. Individuals with PD showed significantly higher levels of depression and stress; and individuals with MS presented significantly higher depression and stress levels, as well as significantly lower quality of life when compared to healthy controls. Further studies are needed to investigate the impact of the COVID-19 pandemic in individuals with AD.

## Introduction

Coronavirus disease 2019 (COVID-19) is an infectious disease caused by severe acute respiratory syndrome coronavirus 2 (SARS-CoV-2). On January 30, 2020, the World Health Organization declared COVID-19 an international public health emergency quickly escalating to a global pandemic on March 11, 2020 [1, 2]. Individuals with chronic diseases are the most vulnerable to infectious diseases such as COVID-19 [3]. Among the chronic diseases that affect the central nervous system, Parkinson's disease (PD), Multiple Sclerosis (MS) and Alzheimer's disease (AD) are the most common [4]. These diseases present varied epidemiology, clinical symptomatology, laboratory and neuroimaging characteristics, neuropathology and management [5].

The neuropathology of PD is characterized by the progressive loss of dopaminergic neurons of the substantia nigra in the midbrain [6]. MS is a chronic immuno-mediated inflammatory condition that affects the central nervous system as a consequence of the infiltration of self-reactive lymphocytes into the blood brain barrier, causing local inflammation that results in demyelination, glial scar formation and axonal loss [7]. AD is marked by the formation of beta-amyloid protein plaques and tangles of tau proteins in neurons located in the brain [8]. It is important to highlight that the neuropathology underlying these conditions has been associated to a greater vulnerability to SARS-CoV-2 infection and the development of COVID-19 [9].

Despite the differences in the underlying neuropathology, these conditions share common characteristics such as the presence of neuropsychiatric disorders (anxiety, depression, and stress), sleep disorders (bad sleep and insomnia) and low quality of life [10–12]. These propensities were further exacerbated during the COVID-19 pandemic with healthcare efforts being shifted from treating chronic illness towards prevention and management of SARS-CoV-2; consequently, having a negative impact on their mental health [13, 14].

The shift observed in the healthcare system increased the likelihood for these individuals to develop, relapse or aggravate neuropsychiatric and sleep disorders potentially leading to a lower quality of life. Abasiyanik, Kurt and Kahraman (2022) have contributed to furthering the scientific knowledge surrounding the impact of COVID-19 on various neurological conditions within their systematic review [15]. Though, a few notable limitations were present within this review such as: quality of evidence and strength of the recommendation were not assessed; a meta-analysis was not performed making effect size estimation challenging; and the clinical heterogeneity between the observational studies was not reported.

To encourage evidence-based practice in informed decision-making regarding the COVID-19 pandemic in individuals with PD, MS, and AD, additional systematic review studies with more rigorous methodology are needed. Therefore, the purpose of this systematic review and meta-analysis was to investigate the impact of the COVID-19 pandemic on 1) neuropsychiatric disorders (depression, anxiety, and stress), 2) sleep disorders (sleep quality and insomnia) and 3) quality of life in individuals with PD, MS and AD compared to healthy controls.

## Methods

This systematic review and meta-analysis followed the Preferred Reporting Items for Systematic Reviews and Meta Analyses (PRISMA) guidelines and the recommendations from the Cochrane Collaboration [16, 17]. The quality of evidence and strength of the recommendation was assessed by the Grading of Recommendations Assessment, Development, and Evaluation (GRADE) approach [18]. The protocol was registered in the International Prospective Register of Systematic Reviews (PROSPERO) (CRD42021286219).

### Search strategy

The electronic search was performed in seven databases (Medline, Embase, ScienceDirect, Web of Science, The Cochrane Library, Scielo and Lilacs) between March 2020 and December 2022, using the following combination of keywords: (Parkinson Disease OR Alzheimer Disease OR Multiple Sclerosis) AND (depression OR anxiety OR stress OR sleep OR insomnia OR quality of life) AND (COVID-19). The search string was composed of keywords selected from the Medical Subjects Headings controlled vocabulary.

### Eligibility criteria

Observational studies (i.e., cross-sectional, case–control, and cohort) were included. For the inclusion criteria, the patient, intervention, comparison, and outcome (PICO) strategy [19, 20] was utilized being 1) (P) Patient: individuals with neurodegenerative and demyelinating diseases (PD, MS, and AD); 2) (I) Intervention: studies conducted during the COVID-19 pandemic regardless of peak contamination, social distancing and quarantine; 3) (C) Comparison: healthy controls, and 4) (O) Outcomes: neuropsychiatric disorders (depression, anxiety, and stress), sleep disorders (sleep quality, and insomnia), and quality of life. Studies using evaluation scales with confirmed psychometric properties (reliability, validity or responsiveness) were included and domain or total scores of these scales were considered. Only full publications in English, Spanish and Portuguese were included. Exclusion criteria included 1) studies that used qualitative data, 2) control groups with other neurodegenerative and/or neurological diseases, 3) studies comparing specific moments of the COVID-19 pandemic (before and during), and 4) intragroup or association analyses (regression).

### Selection process

Title, abstract and full text screenings were conducted by two independent reviewers (M. P. B. O. and C. R. L.) following the inclusion and exclusion criteria. The reference lists of included studies were manually searched to identify possible titles not recovered in the initial electronic searches. Any disagreements in the selection process were resolved by consensus. When consensus was not reached, a third reviewer (S. M. S.) was consulted. The State of the Art through Systematic Review reference manager software was used for the screening process and title selection [21].

### Data extraction

An adapted form from the Cochrane Collaboration was implemented for data extraction of the included studies [17]. Data variables included the study period, data collection procedures, recruitment of individuals, country, and continent. For the neurodegenerative and demyelinating disease group, the type of population, diagnostic criteria, sample size, mean age, and gender were extracted. For the healthy control group, sample size, mean age, and gender were extracted. The outcomes, evaluation scales, and statistical results (*p*-value) of the studies were also recorded. Effect sizes and 95% confidence intervals (CI) were calculated considering the sample size, mean, and standard deviation values [22].

### Quality of evidence and strength of the recommendation assessment: GRADE

The GRADE approach was used to assess the quality of evidence and strength of the recommendation [18]. This approach considers five criteria: limitations (risk of bias), inconsistency, indirectness, imprecision, and publication bias. The studies by comparison group met the limitations criterion when they reached ≥ 9 points (≥ 66.8%) of the total score on the custom checklist of Downs and Black [23, 24]. The interpretation of these criteria allows classifying the evidence as high, moderate, low or very low. It is important to highlight that when observational studies are considered for evidence synthesis, the quality of evidence begins as low [25].

### Data analysis

The studies included in the meta-analysis were grouped based on the outcomes evaluated. The effect size was calculated using standardized mean differences (SMD) with 95% CI. Mean and standard deviation values were used. The effect size was considered significant when the *p-value* was less than 0.05 (*p* < 0.05) and classified as small (< 0.20), moderate (between 0.21 and 0.79) or large (> 0.80) effect size based on Cohen's criteria [26]. Given the variability of the effect size among the included studies, the random effects model was used. Index *I*^*2*^ was used to evaluate the heterogeneity among the studies included in the meta-analysis and interpreted as small (≤ 25%), moderate (between 26 and 74%) or large (≥ 75%) [27]. The statistical tools of the Review Manager software (RevMan version 5.4.1) were used.

### Risk of bias assessment: customized downs and black checklist

A customized checklist was developed to assess the risk of bias of the included studies through the scale proposed by Downs and Black [23]. This scale was developed to assess randomized and non-randomized studies. The original version has five domains and 27 items: reporting (1–10), external validity (11–13), internal validity—bias (14–20), internal validity—selection bias (21–26) and power (27). The customized Downs and Black checklist of this systematic review and meta-analysis was developed including four domains and 13 items: reporting (1–3, 6, 7, 9, 10), external validity (11), internal validity—bias (18, 20), internal validity—selection bias (21, 22, 26). The remaining items on the checklist did not apply to observational studies and therefore were excluded. The responses on the 13 selected items were scored as 0 (no or unable to determine) or 1 (yes) based on the original version with the maximum possible score being 13. Risk of bias associated with each study was classified as low (≥ 9 points [≥ 66.8%]), moderate (between 5 and 8 points (33.4 to 66.7%) or high (≤ 4 points [≤ 33.3%]) by two independent reviewers (A. E. F. C. and A. L. M.) with a third reviewer (M. P. B. O.) being consulted in case of disagreements. This model of personalization and classification of the risk of bias has been previously reported in a systematic review and meta-analysis in individuals with PD [24].

## Results

**Fig. 1:**
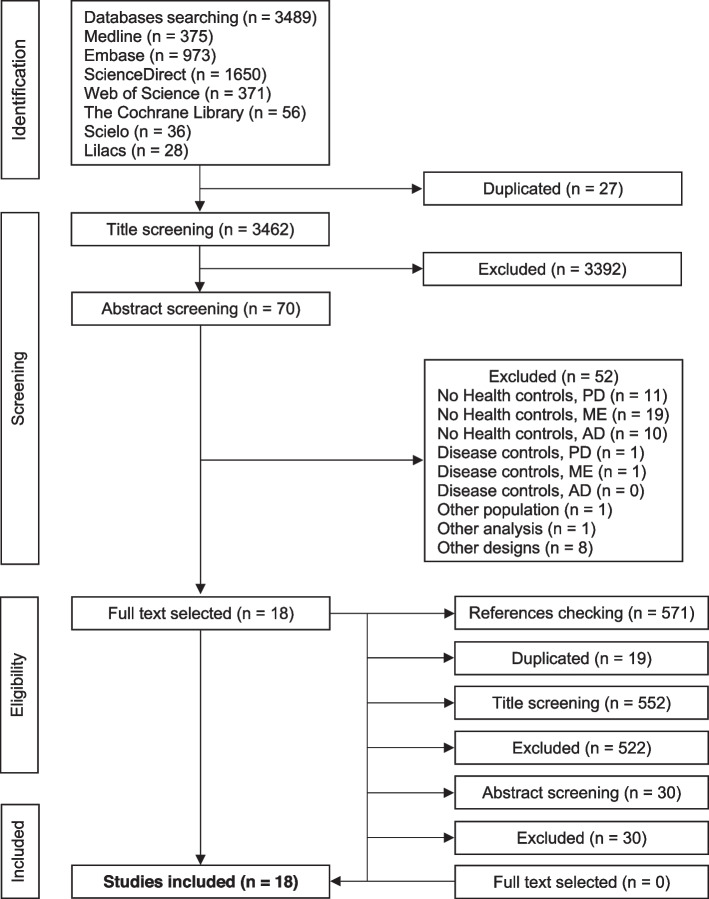
Flow diagram of studies selection process. (PD) Parkinson’s disease; (MS) Multiple Sclerosis; (AD) Alzheimer’s disease

The initial electronic search retrieved 3489 references with no additional studies being retrieved via manual search. After the screening process, 18 studies were included. Seven studies in individuals with PD [28–34] and 11 studies in individuals with MS were identified [35–45]. No study was identified in individuals with AD. Twelve studies (PD = four and MS = 8) were included in the meta-analysis. The study selection flowchart of the systematic review and meta-analysis is presented in Fig. 1.

Fifteen studies were conducted in 2020 (March to December) [30–43, 45], a study in 2021 (January to February) [44] and two studies did not report this information [28, 29]. Data collection procedures were performed by phone, e-mail or online in 14 studies [28–33, 35, 37–41, 43, 45]; in person interviews in one study [44]; and three studies did not report this information [34, 36, 42]. Fifteen studies recruited individuals in specific sites (hospitals, laboratory databases, clinics or treatment centers) [28–37, 40–42, 44, 45] and three studies in non-specific web-based sites (social networks [e.g., facebook or Instagram] and personal communications) [38, 39, 43]. The studies were conducted in 10 different countries (China, Egypt, Italy, Iran, Japan, New Zealand, Republic of Serbia, Turkey, United Kingdom and United States) covering five continents (Africa, Asia, Europe, North America and Oceania). Table 1 presents the main characteristics of the included studies.

**Table 1:** Characteristics of included studies

Authors (year)	Study period Data collection procedure Recruitment of participants Country Continent(s)	Diseases Population Diagnostic criteria Sample size, n Age, mean Sex, female / male	Health controls Sample size, n Age, mean Sex, female / male	(1) Neuropsychiatric disorders, (2) Sleep disorders and (3) Quality of life Outcome(s) / Tool(s) / *p*-value / Effect size (95% confidence interval)
Balci et al. 2021 [31]	Between March to June 2020 Phone Laboratory database Turkey Europe and Asia	PD UKPDSBB 45 67.0 years 15 female; 30 male	43 66.0 years 19 female; 24 male	(1) Depression / HADS-*depression* / *p* 0.524 / - (1) Anxiety / HADS-*anxiety* / *p* 0.977 / -
				(2) -
				(3) -
Blakemore et al. 2021 [32]	Between April to May 2020 Phone or online Laboratory database (New Zealand Brain Research Institute) New Zealand Oceania	PD - 149 72.0 years -	51 78.0 years -	(1) Stress / PSS / *p* > 99% / 0.55 (0.23 to 0.88)
				(2) -
				(3) -
Kitani-Morii et al. 2021 [33]	Between April to May 2020 Phone or e-mail University Hospital, Kyoto Prefectural University of Medicine Japan Asia	PD - 39 72.3 years 14 female; 25 male	32 66.4 years 27 female; 5 male	(1) Depression / PHQ / *p* 0.010 / - (1) Anxiety / GAD / *p* 0.130 / -
				(2) Insomnia / ISI / *p* 0.170 / -
				(3) -
Suzuki et al. 2021 [34]	Between June to December 2020 - Dokkyo Medical University Hospital Japan Asia	PD MDS 100 72.2 years 55 female, 45 male	100 65.5 years 53 female; 47 male	(1) Depression / HADS-*depression* / *p* 0.585 / - (1) Anxiety / HADS-*anxiety* / *p* 1.000 / -
				(2) -
				(3) Quality of life / SF-8-*physical function* / *p* < 0.001 / -0.63 (-0.92 to -0.35); SF-8-*role physical* / *p* < 0.001 / -0.54 (-0.82 to -0.26); SF-8-*bodily pain* / *p* 0.055 / -0.27 (-0.55 to 0.01); SF-8-*general health* / *p* 0.001 / -0.49 (-0.78 to -0.21); SF-8-vitality / *p* < 0.001 / -0.61 (-0.90 to -0.33); SF-8-*social functioning* / *p* 0.949 / -0.01 (-0.29 to 0.27); SF-8-*role emotional* / *p* 0.490 / -0.09 (-0.37 to 0.19); SF-8-*mental health* / *p* 0.804 / -0.04 (-0.32 to 0.23); SF-8-*physical component* / *p* < 0.001 / -0.72 (-1.00 to -0.43); SF-8-*mental component* / *p* 0.349 / 0.13 (-0.15 to 0.40)
Salari et al. 2020 [28]	- Online Movement Disorders Center Iran Asia	PD - 137 55.0 years 90 female; 47 male	442 - -	(1) Anxiety / BAI-II / *p* < 0.001 / 1.04 (0.84 to 1.24)
				(2) -
				(3) -
Shalash et al. 2020 [29]	- Phone Ain Shams University Hospitals Egypt Africa and Asia	PD - 38 55.6 years 9 female; 29 male	20 55.5 years 6 female; 14 male	(1) Depression / DASS-*depression* / *p* 0.015 / 0.68 (0.12 to 1.24) (1) Anxiety / DASS-*anxiety* / *p* 0.001 / 0.84 (0.27 to 1.40) (1) Stress / DASS-*stress* / *p* 0.028 / 0.62 (0.06 to 1.17)
				(2) -
				(3) Quality of life / PDQ-39 / *p* < 0.001 / 0.85 (0.29 to 1.42)
Xia et al. 2020 [30]	April 2020 Online Neurology clinic in Wuhan China Asia	PD UKPDSBB 119 61.2 years 58 female; 61 male	169 59.8 years 93 female; 76 male	(1) Depression / HADS-*depression* / *p* 0.022 / 0.27 (0.03 to 0.51) (1) Anxiety / HADS-*anxiety* / *p* 0.579 / 0.07 (-0.16 to 0.31)
				(2) Sleep quality / PSQI / *p* < 0.001 / 0.74 (0.50 to 0.98)
				(3) -
Goverover et al. 2022 [43]	Between July to October 2020 Online Web-based (unspecific) United States North America	MS - 69 48.1 years - (unclear information)	95 42.9 years 79 female; 16 male	(1) -
				(2) -
				(3) Quality of life / FACT-7 / p < 0.001 / 1.07 (0.74 to 1.40)
Koc et al. 2022 [44]	Between January to February 2021 Face-to-face interviews Neurology Clinic of Uludag University, Faculty of Medicine Turkey Europe and Asia	MS - 86 38.1 years 57 female; 29 male	65 38.0 years 43 femanle; 22 male	(1) Depression / BDI / *p* < 0.001 / - (1) Anxiety / BAI / *p* 0.010 / -
				(2) Sleep quality / PSQI / *p* 0.731 / -
				(3) Quality of life / SF-36-*physical functioning* / *p* < 0.001 / -; SF-36-*physical role limitations* / *p* 0.001 / -; SF-36-*emotional role limitations* / *p* 0.080 / -; SF-36-*vitality* / *p* 0.010 / -; SF-36-*emotional well-being* / *p* 0.055 / -; SF-36-*social functioning* / *p* 0.650 / -; SF-36-*pain* / *p* 0.128 / -; SF-36-*general health* / *p* < 0.001 / -
Yeni, Tulek and Terzi, 2022 [45]	December 2020 Online Neurology outpatient clinic of a University Hospital Turkey Europe and Asia	MS - 89 41.1 years 56 female; 33 male	262 38.1 years 135 female; 127 male	(1) Depression / BDI / *p* 0.001 / 0.39 (0.14 to 0.63) (1) Anxiety / WAQ / *p* 0.001 / 0.35 ( 0.11 to 0.59)
				(2) Sleep quality / PSQI / *p* 0.906 / 0.01 (-0.23 to 0.25)
				(3) -
Bonavita et al. 2021 [38]	April 2020 Online Web-based (unspecific) Italy Europe	MS - 612 43.0 years 465 female; 147 male	674 44.0 years 490 female; 184 male	(1) Depression / PHQ / *p* < 0.001 / - (1) Stress / PSS / *p* < 0.001 / -
				(2) -
				(3) -
Costabile et al. 2021 [39]	Between April to May 2020 Online Web-based (unspecific) Italy Europe	MS - 497 42.4 years 351 female; 146 male	348 40.8 years 264 female; 84 male	(1) -
				(2) -
				(3) Quality of life / NeuroQoL-*cognitive disfunction* / *p* < 0.0001 / 0.50 (0.37 to 0.64); NeuroQoL-*abstraction and logical abilities* / *p* 0.1 / -0.34 (-0.48 to -0.21); NeuroQoL-*depression* / *p* 0.005 / 0.25 (0.12 to 0.39); NeuroQoL-*anxiety* / *p* 0.064 / 0.20 (0.06 to 0.34); NeuroQoL-*emotional dyscontrol* / *p* 0.015 / 0.21 (0.07 to 0.35); NeuroQoL-*sleep disturbances* / p 0.017 / 0.22 (0.08 to 0.36)
Garjani et al. 2021 [40]	Between May to July 2020 Online Laboratory database (United Kingdom MS Register) United Kingdom Europe	MS - 2010 56.0 years 1488 female; 522 male	380 49.0 years 248 female; 132	(1) Depression / PHQ / *p* 0.002 / - (1) Anxiety / GAD / *p* 0.081 / - (1) Stress / IES-R / *p* 0.52 / -
				(2) -
				(3) -
Shaygannejad, Afshari-Safavi and Hatef, 2021 [41]	Between March to April 2020 Online Kashani Hospital Iran Asia	MS McDonald’s 223 35.9 years 183 female; 40 male	245 34.2 years 185 female; 60 male	(1) Depression / DASS-*depression* / *p* 0.054 / 0.36 (0.17 to 0.54) (1) Anxiety / DASS-*anxiety* / *p* 0.080 / 0.36 (0.18 to 0.54) (1) Stress / DASS-*stress* / *p* 0.011 / 0.38 (0.20 to 0.56)
				(2) -
				(3) -
Stojanov et al. 2021 [42]	Between April to May 2020 - Laboratory database Republic of Serbia Europe	MS McDonald’s 67 45.1 years 45 female; 22 male	85 44.2 years 56 female; 29 male	(1) -
				(2) Sleep quality / PSQI / *p* < 0.01 / 1.74 (1.36 to 2.12)
				(3) -
Motolese et al. 2020 [35]	Between April to May 2020 Online Policlinico Universitario Campus Bio-Medico Italy Europe	MS - 60 < 50 years (n = 40); > 50 years (n = 20) 41 female; 19 male	50 < 50 years (n = 34); > 50 years (n = 16) 31 female; 19 male	(1) Depression / BDI-*total* / *p* 0.010 / 0.33 (-0.05 to 0.70); BDI-*neuroveg* / *p* 0.006 / 0.44 (0.06 to 0.82); BDI-*cognitive* / *p* 0.152 / 0.26 (-0.12 to 0.64) (1) Anxiety / GAD / *p* 0.0331 / -0.31 (-0.69 to 0.07)
				(2) Sleep quality / PSQI / *p* 0.001 / 0.67 (0.28 to 1.05)
				(3) -
Stojanov et al. 2020 [36]	April 2020 - Laboratory database Republic of Serbia Europe	MS McDonald’s 95 43.4 years 64 female; 31 male	99 44.3 years 66 female; 33 male	(1) Depression / HAM-D / *p* < 0.01 / 2.08 (1.73 to 2.43) (1) Anxiety / HAM-A / *p* < 0.01 / 1.84 (1.50 to 2.18)
				(2) -
				(3) Quality of life / MSQoL-54-*mental health* / *p* < 0.01 / -1.57 (-1.89 to -1.25); MSQoL-54-*physical health* / *p* < 0.01 / -2.31 (-2.67 to -1.94)
Talaat et al. 2020 [37]	Between March to April 2020 Online Alexandria University Hospital Egypt Africa and Asia	MS McDonald’s 115 34.4 years 89 female; 26 male	129 31.7 years 93 female; 36 male	(1) Depression / DASS-*depression* / *p* 0.001 / 0.54 (0.28 to 0.79) (1) Anxiety / DASS-*anxiety* / *p* < 0.001 / 0.40 (0.15 to 0.66) (1) Stress / DASS-*stress* / *p* < 0.001 / 1.01 (0.75 to 1.28)
				(2) -
				(3) -

*PD* Parkinson’s disease, *MS* Multiple Sclerosis, *MDS* Movement Disorder Society, *UKPDSBB* United Kingdom Parkinson’s Disease Society Brain Bank, *n* sample size, *BAI* Beck Anxiety Inventory-II, *BDI* Beck Depression Inventory, *DASS* Depression, Anxiety, and Stress Scale, *FACT-7* Functional Assessment of Cancer Therapy-7, *GAD* Generalized Anxiety Disorder, *HADS* Hospital Anxiety and Depression Scale, *HAM-A* Hamilton Anxiety Scale, *HAM-D* Hamilton Depression Scale, *IES-R* Impact of Event Scale-Revised, *ISI* Insomnia Severity Index, *MSQoL-54* Multiple Sclerosis Quality of Life-54 Instrument, *NeuroQoL* Quality of Life in Neurological Disorders, *PDQ-39* Parkinson's Disease Questionnaire-39, *PHQ* Patient Health Questionnaire, *PSQI* Pittsburgh Sleep Quality Index, *PSS* Perceived Stress Scale, *SF-8* Short Form of Quality of Life-8, *SF-36* Short Form of Quality of Life-36, *WAQ* Worry and Anxiety Questionnaire

Seven studies were performed in individuals with PD [28–34]. Of these, three studies defined the diagnostic criterion with the two studies [30, 31] using the United Kingdom Parkinson’s Disease Society Brain Bank [46] and one study [34] using the Movement Disorder Society [47]. The remaining four studies did not report this information [28, 29, 32, 33]. The selected studies totaled a sample of 627 individuals with PD. The mean age was 65.0 (range 55.0 to 72.3). According to the studies that reported biological sex as a variable, 241 of 478 individuals were female (50.4%) and 237 were male (49.6%). One study did not report biological sex of its participants [32]. Regarding healthy controls, a total of 857 individuals were used as comparators. According to the studies that reported age (*n* = 413) and biological sex (*n* = 364) as a variable, the mean age was 65.2 years (range 55.5 to 78) with 198 being female (54.4%) and 166 being male (45.6%). One study did not report the mean age [28] and two studies did not report the biological sex of its participants [28, 32].

Eleven studies were performed in individuals with MS [35–45]. Four studies [36, 37, 41, 42] defined the diagnostic criterion according to the McDonald's criteria [48]. Seven studies did not report this information [35, 38–40, 43–45]. The selected studies totaled a sample of 3923 individuals with MS. Of 3863 individuals, the mean age was 42.7 (range 34.4 to 48.1). One study did not report the mean age of the individuals [35]. Of 3854 individuals, 2839 were women (73.7%) and 1015 were men (26.3%). In another study, information on the biological sex of individuals was not available [43]. Regarding healthy controls, 2432 individuals were used as comparators. Of 2382 individuals, the mean age was 40.7 (range 31.7 to 44.3). One study did not report the mean age of its participants [35]. From a total of 2432 individuals, 1690 were female (69.5%) and 742 male (30.5%).

Fifteen studies investigated neuropsychiatric disorders (PD = seven [28–34]; MS = eight [35–38, 40, 41, 44, 45]). Thirteen studies investigated depression (PD = five [29–31, 33, 34]; and MS = eight [35–38, 40, 41, 44, 45]). The following measures were implemented to assess depression in individuals with PD: 1) Depression, Anxiety, and Stress Scale (DASS) (depression domain), 2) Hospital Anxiety and Depression Scale (HADS) (depression domain), and 3) Patient Health Questionnaire (PHQ); and in individuals with MS: 1) Beck Depression Inventory, 2) DASS (depression domain), 3) Hamilton Depression Scale, and 4) PHQ. Thirteen studies investigated anxiety (PD = six [28–31, 33, 34] and MS = seven [35–37, 40, 41, 44, 45]). The following measures were implemented to assess depression in individuals with PD: 1) Beck Anxiety Inventory (BAI), 2) DASS (anxiety domain), 3) Generalized Anxiety Disorder (GAD), and 4) HADS (anxiety domain); and in individuals with MS: 1) BAI, 2) DASS (anxiety domain), 3) GAD, 4) Hamilton Anxiety Scale, and 5) Worry and Anxiety Questionnaire. Six studies investigated stress (PD = two [29, 32] and MS = four [37, 38, 40, 41]). Studies in individuals with PD assessed stress via 1) DASS (stress domain), and 2) Perceived Stress Scale (PSS); and studies in individuals with MS assessed stress via 1) DASS (stress domain), 2) Impact of Event Scale-Revised, and 3) PSS.

Six studies investigated sleep disorders (PD = two [30, 33] and MS = four [35, 36, 44, 45]). Five studies analyzed sleep quality (PD = one [30] and MS = four [35, 42, 44, 45]) through the Pittsburgh Sleep Quality Index. One study analyzed insomnia in individuals with PD and used the Insomnia Severity Index [33]. It was not possible to synthesize scientific evidence for insomnia based on the results.

Six studies investigated quality of life (PD = two [29, 34] and MS = four [36, 39, 43, 44]). The Parkinson's Disease Questionnaire-39 and Short Form of Quality of Life-8 were used to assess quality of life in individuals with PD; and the Functional Assessment of Cancer Therapy-7, Quality of Life in Neurological Disorders, Multiple Sclerosis Quality of Life-54 Instrument, and Short Form of Quality of life-36 for individuals with MS.

### Quality of evidence and strength of the recommendation, and data analysis

**Fig. 2:**
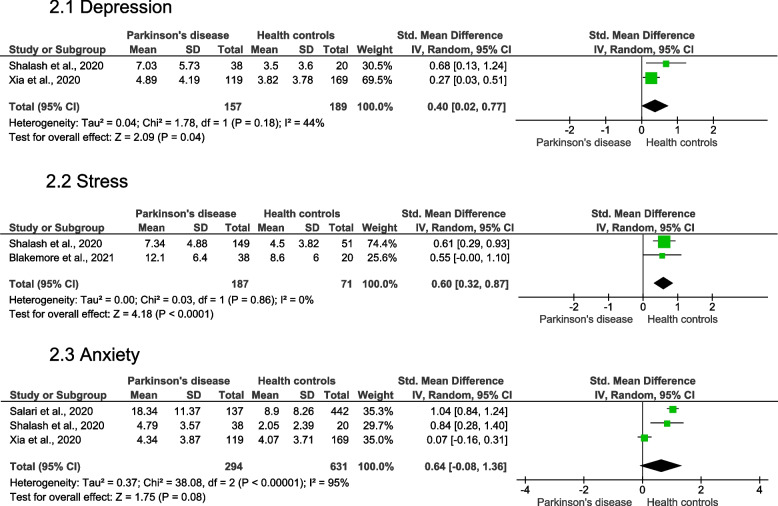
The forest plots for the grouped studies in individuals with Parkinson's disease for depression, stress, and anxiety

Individuals with PD presented significantly higher levels of depression (very low quality of evidence [downgraded for indirectness], SMD = 0.40 [moderate effect], 95% CI = 0.02 to 0.77, *p* = 0.04 and* I*^*2*^ = 44% [moderate heterogeneity]) (Fig. 2.1) and stress (very low quality of evidence [downgraded for indirectness], SMD = 0.60 [moderate effect], 95% CI = 0.32 to 0.87,* p* < 0.0001 and* I*^*2*^ = 0% [low heterogeneity]) (Fig. 2.2). No significant changes were observed for the anxiety (very low quality of evidence [downgraded for limitations (risk of bias), inconsistency and indirectness], SMD = 0.64 [moderate effect], 95% CI = -0.08 to 1.36, *p* = 0.08 and* I*^*2*^ = 95% [high heterogeneity]) (Fig. 2.3). The forest plots for the grouped studies in individuals with PD is shown in Fig. 2.

**Fig. 3:**
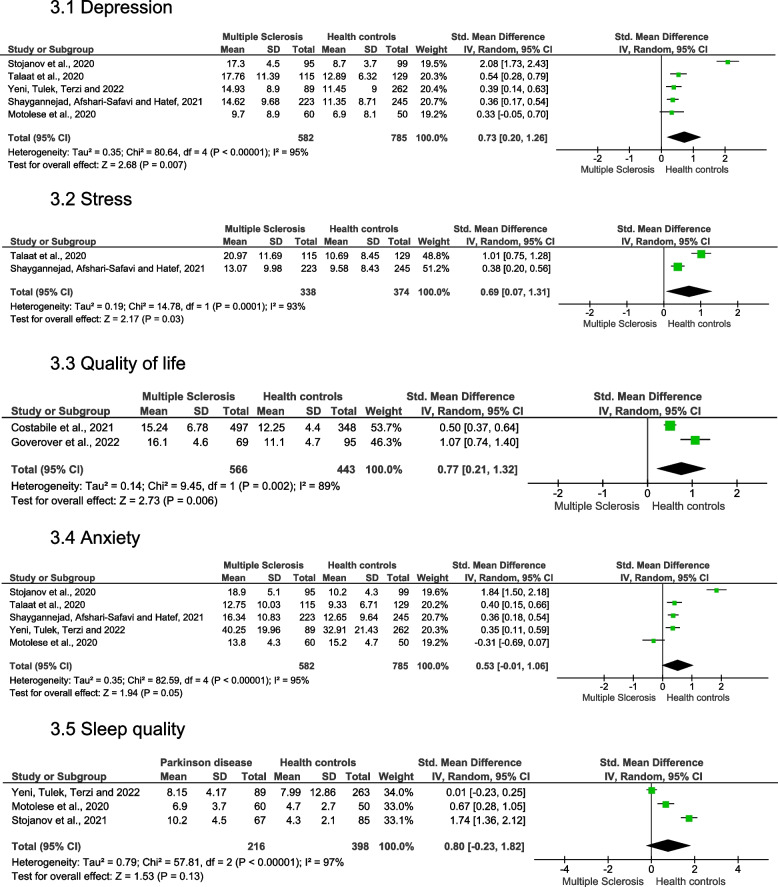
The forest plots for the grouped studies in individuals with Multiple Sclerosis for depression, stress, quality of life, anxiety, and sleep quality

Individuals with MS showed significantly higher levels of depression (very low quality of evidence [downgraded for indirectness], SMD = 0.73 [moderate effect], 95% CI = 0.20 to 1.26,* p* = 0.007 and* I*^*2*^ = 95% [high heterogeneity]) (Fig. 3.1), higher levels of stress (low quality of evidence, SMD = 0.69 [moderate effect], 95% CI = 0.07 to 1.31,* p* = 0.03 and* I*^*2*^ = 93% [high heterogeneity]) (Fig. 3.2), and lower quality of life (very low quality of evidence [downgraded for indirectness], SMD = 0.77 [moderate effect], 95% CI = 0.21 to 1.32,* p* = 0.006 and* I*^*2*^ = 89% [high heterogeneity]) (Fig. 3.3). No significant changes were observed for the anxiety (very low quality of evidence [downgraded for indirectness], SMD = 0.53 [moderate effect], 95% CI = -0.01 to 1.06, *p* = 0.05 and* I*^*2*^ = 95% [high heterogeneity]) (Fig. 3.4) and sleep quality (low quality of evidence, SMD = 0.80 [large effect], 95% CI = -0.23 to 1.82, *p* = 0.13 and *I*^*2*^ = 97% [high heterogeneity]) (Fig. 3.5). The forest plots for the grouped studies in individuals with MS is shown in Fig. 3. The quality of evidence and strength of recommendation, and the interpretation of the GRADE domains are presented in Table 2.

**Table 2:** Overview of the GRADE approach

Outcomes	(1)	(2)	(3)	(4)	(5)	Studies	N° of participants (Diseases *vs* Health controls) / N° of studies		^*^Effect size	Quality of the evidence
Parkinson’s disease										
Depression	Ns	Ns	S^C^	Ns	Ns	Shalash et al. 2020 [29] Xia et al. 2020 [30]	157	189	SMD = 0.40 (moderate effect) 95% CI = 0.02 to 0.77 *p* = 0.04 *I*^*2*^ = 44% (moderate heterogeneity)	⨁⨁⨁◯ Very low
							(two studies)			
Stress	Ns	Ns	S^C^	Ns	Ns	Blakemore et al. 2021 [32] Shalash et al. 2020 [29]	187	71	SMD = 0.60 (moderate effect) 95% CI = 0.32 to 0.87 *p* < 0.0001 *I*^*2*^ = 0% (low heterogeneity)	⨁⨁⨁◯ Very low
							(two studies)			
Anxiety	S^A^	S^B^	S^C^	Ns	Ns	Salari et al. 2020 [28] Shalash et al. 2020 [29] Xia et al. 2020 [30]	294	631	SMD = 0.64 (moderate effect) 95% CI = -0.08 to 1.36 *p* = 0.08 *I*^*2*^ = 95% (high heterogeneity)	⨁◯◯◯ Very low
							(three studies)			
Multiple Sclerosis										
Depression	Ns	Ns	S^C^	Ns	Ns	Yeni, Tulek and Terzi, 2022 [45] Shaygannejad, Afshari-Safavi and Hatef, 2021 [41] Motolese et al. 2020 [35] Stojanov et al. 2020 [36] Talaat et al. 2020 [37]	582	785	SMD = 0.73 (moderate effect) 95% CI = 0.20 to 1.26 *p* = 0.007 *I*^*2*^ = 95% (high heterogeneity)	⨁⨁⨁◯ Very Low
							(five studies)			
Stress	Ns	Ns	Ns	Ns	Ns	Shaygannejad, Afshari-Safavi and Hatef, 2021 [41] Talaat et al. 2020 [37]	338	334	SMD = 0.69 (moderate effect) 95% CI = 0.07 to 1.31 *p* = 0.03 *I*^*2*^ = 93% (high heterogeneity)	⨁⨁⨁⨁ Low
							(two studies)			
Quality of life	Ns	Ns	S^C^	Ns	Ns	Goverover et al. 2022 [43] Costabile et al. 2021 [39]	566	443	SMD = 0.77 (moderate effect) 95% CI = 0.21 to 1.32 *p* = 0.006 *I*^*2*^ = 89% (high heterogeneity)	⨁⨁⨁◯ Very Low
							(two studies)			
Anxiety	Ns	Ns	S^C^	Ns	Ns	Yeni, Tulek and Terzi, 2022 [45] Shaygannejad, Afshari-Safavi and Hatef, 2021 [41] Motolese et al. 2020 [35] Stojanov et al. 2020 [36] Talaat et al. 2020 [37]	582	785	SMD = 0.53 (moderate effect) 95% CI = -0.01 to 1.06 *p* = 0.05 *I*^*2*^ = 95% (high heterogeneity)	⨁⨁⨁◯ Very Low
							(five studies)			
Sleep quality	Ns	Ns	Ns	Ns	Ns	Yeni, Tulek and Terzi, 2022 [45] Stojanov et al. 2021 [42] Motolese et al. 2020 [35]	216	397	SMD = 0.80 (large effect) 95% CI = -0.23 to 1.82 *p* = 0.13 *I*^*2*^ = 97% (high heterogeneity)	⨁⨁⨁⨁ Low
							(three studies)			

*SMD* standardized mean differences, *95% CI* 95% of confidence interval, *I2* consistency between the studies

(1) Limitations (risk of bias); (2) Inconsistency; (3) Indirectness; (4) Imprecision; (5) Publication bias; (Ns) No serious; (S) Serious; A Downgrading due to < 75% of the studies presented low risk of bias; B Downgrading due to different directions towards the results found; C Downgrading due to heterogeneity of the scales for evaluating measures of results; D Downgrading due to was sparse data with less than 200 individuals per comparison; E Downgrading if > 50% of the studies are from the same research team

^*^Cohen's criteria: small effect (< 0.20), moderate effect (between 0.21 and 0.79) or large effect (> 0.80)

### Risk of bias

The final score for the 18 studies included in the customized checklist of Downs and Black ranged from eight [28] to 12 [31]. The average score was 10.5. Seventeen studies had a low risk of bias (94.4%). Three items presented percentages below 75% and were the least attended (3, 9, and 26). Of the 18 studies included, 13 (27.8%) did not meet item 26 (internal validity), 11 (38.9%) did not meet item 3 (reporting—patients included), and 11 (38.9%) did not meet item 9 (reporting—patients lost). The score for the 18 included studies is presented in Table 3.

**Table 3:** Customized downs and black checklist for bias risk assessment

Author (years)	**Reporting**							**External Validity**	**Internal Validity (bias)**		**Internal Validity—Confounding (selection bias)**			
	(1)	(2)	(3)	(6)	(7)	(9)	(10)	(11)	(18)	(20)	(21)	(22)	(26)	Total score
Balci et al. 2021 [31]	1	1	1	1	1	0	1	1	1	1	1	1	1	*12/13 92.3%
Koc et al. 2022 [44]	1	1	1	1	1	0	1	1	1	1	1	1	0	*11/13 84.6%
Yeni, Tulek and Terzi, 2022 [45]	1	1	0	1	1	0	1	1	1	1	1	1	1	*11/13 84.6%
Blakemore et al. 2021 [32]	1	1	0	1	1	1	1	1	1	1	1	1	0	*11/13 84.6%
Bonavita et al. 2021 [38]	1	1	0	1	1	1	1	1	1	1	0	1	1	*11/13 84.6%
Costabile et al. 2021 [39]	1	1	0	1	1	1	1	1	1	1	0	1	1	*11/13 84.6%
Garjani et al. 2021 [40]	1	1	0	1	1	1	1	1	1	1	1	1	0	*11/13 84.6%
Kitani-Morii et al. 2021 [33]	1	1	0	1	1	1	1	1	1	1	1	1	0	*11/13 84.6%
Suzuki et al. 2021 [34]	1	1	1	1	1	0	1	1	1	1	1	1	0	*11/13 84.6%
Motolese et al. 2020 [35]	1	1	0	1	1	1	1	1	1	1	1	1	0	*11/13 84.6%
Talaat et al. 2020 [37]	1	1	0	1	1	0	1	1	1	1	1	1	1	*11/13 84.6%
Xia et al. 2020 [30]	1	1	1	1	1	1	1	1	0	1	1	1	0	*11/13 84.6%
Shaygannejad, Afshari-Safavi and Hatef, 2021 [41]	1	1	0	1	1	0	1	1	1	1	1	1	0	*10/13 76.9%
Stojanov et al. 2021 [42]	1	1	1	1	1	0	1	1	0	1	1	1	0	*10/13 76.9%
Shalash et al. 2020 [29]	1	1	1	1	1	0	1	1	1	1	1	0	0	*10/13 76.9%
Stojanov et al. 2020 [36]	1	1	1	1	1	0	1	1	0	1	1	1	0	*10/13 76.9%
Goverover et al. 2022 [43]	1	1	0	1	1	0	1	1	1	1	0	1	0	*9/13 69.2%
Salari et al. 2020 [28]	1	1	0	1	1	0	1	1	0	1	1	0	0	8/13 61.5%
Studies which answered the criteria (%)	18/18 100%	18/18 100%	7/18 38.9%	18/18 100%	18/18 100%	7/18 38.9%	18/18 100%	18/18 100%	14/18 77.8%	18/18 100%	15/18 83.3%	16/18 88.9%	5/18 27.8%	

Abbreviation: (0) No / Unable to determine; (1) Yes

(1) Is the hypothesis/aim/objective of the study clearly described? (2) Are the main outcomes to be measured clearly described in the Introduction or Methods section? (3) Are the characteristics of the patients included in the study clearly described? (6) Are the main findings of the study clearly described? (7) Does the study provide estimates of the random variability in the data for the main outcomes? (9) Have the characteristics of patients lost to follow‐up been described? (10) Have actual probability values been reported (e.g., 0.035 rather than < 0.05 for the main outcomes except Where the probability value is less than 0.001? (11) Were the subjects asked to participate in the study representative of the entire population from which they were recruited? (18) Were the statistical tests used to assess the main outcomes appropriate? (20) Were the main outcome measures used accurate (valid and reliable)? (21) Were the patients in different intervention groups (trials and cohort studies) or were the cases and controls (case‐control studies) recruited from the same population? (22) Were study subjects in different intervention groups (trials and cohort studies) or were the cases and controls (case‐control studies) recruited over the same period of time? (26) Were losses of patients to follow‐up taken into account?

^*^High methodological quality (score ≥ 9 [≥ 66.8%])

## Discussion

This systematic review and meta-analysis investigated the impact of the COVID-19 pandemic on neuropsychiatric disorders, sleep disorders, and quality of life in individuals with PD, MS, and AD compared to healthy controls. The results showed the negative repercussion of the COVID-19 pandemic in individuals with PD and MS. Both individuals with PD and MS presented higher levels of depression and stress. Additionally, individuals with MS presented lower quality of life compared to healthy controls. It was not possible to synthesize scientific evidence of the impact of the COVID-19 pandemic in individuals with AD.

### Parkinson’s disease

Individuals with PD had significantly elevated levels of depression during the COVID-19 pandemic compared to healthy control (*p* = 0.04). The quality of the evidence was very low. The size of the effect was moderate (SMD = 0.40). This comparison group presented moderate heterogeneity (*I*^*2*^ = 44%) [29, 30]. It is estimated that about 25% of the meta-analysis present values of* I*^*2*^ above 50% [27]. Even though the heterogeneity of this comparison group was below 50%, this result should be analyzed with caution.

Before the COVID-19 pandemic, depression was the fourth most prevalent neuropsychiatric disorder in individuals with PD when compared to healthy controls (36.6% and 14.9%) [49]. Therefore, the identification of high levels of depression during the COVID-19 pandemic highlights the need for improved clinical management of depression in this population considering that this neuropsychiatric disorder is treatable. The literature shows that pharmacological (selective serotonin reuptake inhibitors) and non-pharmacological (aerobic physical activity and cognitive behavioral therapy) treatments have the potential to alleviate depression in these individuals [50, 51].

On the other hand, individuals with PD did not present high levels of anxiety (*p* = 0.08) during the COVID-19 pandemic compared to healthy controls. The quality of the evidence was very low. A high heterogeneity (*I*^*2*^ = 95%) was identified in this comparison group [28–30]. In part, this high heterogeneity may be due to the different directions of the statistical results and effect sizes observed among the analyzed studies. Additionally, this analysis presented a study with moderate risk of bias potentially influencing the results [28].

Different delivery methods were also implemented for the measures of anxiety (i.e., interview or self-report) which have been previously reported to potentially interfere with anxiety outcomes in individuals with PD [49]. Two studies were delivered online (self-report) [28, 30] and one study by telephone (interview) [29]. One could hypothesize that such differences in the delivery methods observed in the included studies may have influenced the results by the potential underestimation of the participant’s condition in a self-reported method versus a more in-depth analysis when the interview is conducted by a trained evaluator.

Individuals with PD had significantly higher levels of stress during the COVID-19 pandemic compared to healthy controls (*p* < 0.0001). The quality of the evidence was very low with a moderate effect size (SMD = 0.60). This comparison group presented homogeneous results (*I*^*2*^ = 0%) [29, 32]. Stress was evaluated by two different scales (Perceived Stress Scale and Depression, Anxiety, and Stress Scale) in the included studies suggesting that the use of different types of scales to assess stress in these individuals does not seem to interfere with the results.

Stress plays an important role in the development of depression in individuals with PD and has been shown to act as a key element influencing its pathophysiology [52, 53]. Although we cannot establish the relationship between stress and depression during the COVID-19 pandemic in these individuals, it is important to highlight that this systematic review and meta-analysis identified high levels of these two neuropsychiatric disorders. In addition to its contribution to depression in individuals with PD, stress can also contribute to the worsening of motor symptoms with the progression of PD [52]. Therefore, the identification of stress during the COVID-19 pandemic in this population is of great importance.

### Multiple sclerosis

Individuals with MS had significantly elevated levels of depression during the COVID-19 pandemic compared to healthy controls (*p* = 0.007). The quality of the evidence was very low with a moderate effect size (SMD = 0.73). However, a high heterogeneity was identified in this comparison group (*I*^*2*^ = 95%) [35–37, 41, 45]. One study did not observe a significant increase in depression in individuals with MS [41]. Therefore, the different statistical results do not seem to be the main reason for the high heterogeneity. In part, we hypothesize that the observed high heterogeneity may be due to the different scales used to assess depression in this population.

A systematic review and meta-analysis study conducted before the COVID-19 pandemic observed a high prevalence of depression in individuals with MS (30.5%) [54]. A high heterogeneity was identified in this study (*I*^*2*^ = 99.4%). Therefore, it seems that systematic reviews and meta-analysis studies that report higher levels of depression in these individuals also identify high heterogeneity. The identification of high levels of depression during the COVID-19 pandemic in this population is of great importance since psychological and pharmacological treatments for depression in individuals with MS have been shown to be effective in reducing depressive symptoms [55].

On the other hand, individuals with MS did not present high levels of anxiety (*p* = 0.05) during the COVID-19 pandemic compared to healthy controls. The quality of the evidence was very low, and a high heterogeneity was identified in this comparison group (*I*^*2*^ = 95%) [35–37, 41, 45]. It is important to highlight that in an included study, healthy individuals had higher levels of anxiety compared to individuals with MS [35]. In part, we hypothesize that this result may be one of the reasons for the observed high heterogeneity in this comparison group.

Previous literature has shown the high prevalence of anxiety in individuals with MS before the COVID-19 pandemic (22.1%) [54]. However, the same was not identified in our systematic review and meta-analysis during the COVID-19 pandemic. The included studies used four different types of scales to assess anxiety which may have influenced the outcome. A study developed and evaluated the psychometric properties of the specific Coronavirus Anxiety Scale to assess the pandemic-associated anxiety of COVID-19. This scale was shown to be reliable and valid for scientific research and clinical practice [56]. However, to our knowledge, its application has not been tested in individuals with MS and PD. We encourage that future studies in different populations standardize the use of the Coronavirus Anxiety Scale; therefore, facilitating its comparative use across studies.

Individuals with MS had significantly higher levels of stress during the COVID-19 pandemic compared to healthy controls (*p* = 0.03). The quality of the evidence was low with a moderate effect size (SMD = 0.69). It is important to highlight that this comparison group met all the criteria for the GRADE approach reinforcing the quality and strength of the recommendation of this scientific evidence. However, high heterogeneity was identified in this comparison group (*I*^*2*^ = 95%) [37, 41]. The included studies used the Depression, Anxiety, and Stress Scale to assess stress. However, the observed effect sizes varied between large [37] and moderate [41] partly justifying the high heterogeneity.

An additional factor that can justify this high heterogeneity is the temporal structure of MS, that is, whether stress assessment was performed at the beginning or during progressive states of the disease [57]. For this reason, stress is the neuropsychiatric disorder with the greatest observed controversy in studies conducted before the COVID-19 pandemic in individuals with MS [57]. However, there is strong scientific evidence of the association between stress and progression of MS [57], which highlights the importance of identifying the increase in stress during the COVID-19 pandemic in this population. Thus, stress management strategies such as breathing and muscle relaxation techniques that have shown benefits in these individuals should be implemented as part of the treatment strategy [58].

Regarding sleep, individuals with MS did not present low sleep quality during the COVID-19 pandemic compared to healthy controls (*p* = 0.13). The quality of the evidence was low. Although no significant difference in effect size has been identified, this comparison group met all the criteria for the GRADE approach. A high heterogeneity was identified in this comparison group (*I*^*2*^ = 97%) [35, 42, 45]. The studies used the Pittsburgh Sleep Quality Index to assess sleep quality. Although these studies used the same evaluation scale, the direction of statistical results and effect sizes were different, which in part may have contributed to the observed high heterogeneity.

Restless leg syndrome or Willis-Ekbom's disease and sleep apnea were the most investigated sleep disorders in individuals with MS before the COVID-19 pandemic [59]. During the COVID-19 pandemic, sleep quality was investigated in three studies [35, 42, 45] of which none investigated insomnia. This result identifies that some of the major sleep disorders (i.e., insomnia) may have been overlooked in this population during the COVID-19 pandemic. This result was observed by a systematic review study conducted before the COVID-19 pandemic [59]. In addition, it is important to highlight that poor sleep quality is related to lower quality of life observed in this population [60].

Individuals with MS presented lower quality of life during the COVID-19 pandemic compared to healthy controls (*p* = 0.006). The quality of the evidence was very low with a moderate effect size (SMD = 0.77); however, high heterogeneity was identified in this comparison group (*I*^*2*^ = 89%) [39, 43]. Different scales were used to assess quality of life and the effect sizes differed between studies. In part, these methodological and results differences among the studies included in this meta-analysis may justify the observed high heterogeneity.

The World Health Organization during the COVID-19 pandemic instituted a series of strict measures to contain the advance of SARS-CoV-2, such as social distancing and quarantine [1]. These measures caused a broad, substantial, and lasting psychological impact worldwide [61], which may be intrinsically associated with the observed low quality of life of individuals with MS during the COVID-19 pandemic. Furthermore, a systematic review study conducted before the COVID-19 pandemic observed that psychosocial, clinical, and demographic factors are important determinants of low quality of life in these individuals [62], reinforcing the hypothetical association between the healthcare measures implemented to contain the spread of SARS-CoV-2 and lower levels of quality of life in these individuals. Gil-González and colleagues (2020) also identified that among various neuropsychiatric disorders, depression and anxiety were associated with lower quality of life in individuals with MS prior to the COVID-19 pandemic [62]. Therefore, the significantly higher levels of depression found in our analysis may have also contributed to the lower quality of life observed in this population.

### Alzheimer's disease

No study in individuals with AD met the inclusion criteria for this systematic review and meta-analysis. We hypothesize that two factors could be the main drivers for such results. First, even though the impact of COVID-19 has been investigated in individuals with dementia of various etiologies, many of the studies were limited by a lack of comparison to healthy controls. Such limitation makes it difficult to draw conclusions that are specific to the disease being studied which could potentially skew the outcome analysis in this systematic review and meta-analysis. Therefore, studies lacking a control group with healthy participants were excluded. Second, most of the assessments used for neuropsychiatric disorders, sleep disorders and quality of life in individuals with AD rely on the caregiver's report potentially adding an additional layer of subjectivity and hindering the conductance of studies of this nature.

Hughes, Liu and Baumbach (2021) study has reported a negative impact of the COVID-19 pandemic on individuals with dementia [63]. An interruption of daily activities has also been associated with the onset or worsening of neuropsychiatric disorders, such as depression, and anxiety [64]; therefore, it is important that future rigorous studies quantify these disorders in individuals with dementia, including AD to implement strategies that target the symptoms associated with disease progression as well as to investigate their impact on measures of quality of life in this population. In addition, previous evidence suggests the prevalence of depression and anxiety in caregivers of individuals with dementia during the COVID-19 pandemic highlighting the importance of more rigorous studies not only for this population, but for their caregivers as well [63].

## Methodological considerations

Eight scientific measures were synthesized and assessed based on the GRADE approach (PD = three and MS = five). Although the quality of two measures in individuals with MS were low (stress and sleep quality), it is important to highlight that the comparison groups met all the criteria for the GRADE approach. However, the quality of evidence and strength of the recommendation in systematic review studies and meta-analysis of observational studies begin as low quality and not at high quality as observed in randomized clinical trials [25].

In other scientific measures (PD = anxiety, depression, and stress and MS = depression, anxiety, and quality of life), the main criterion responsible for lowering the quality of evidence to “very low” was indirectness related to the heterogeneity of the scales used to evaluate the outcomes. Therefore, we recommend that future studies standardize scales for the evaluation of neuropsychiatric disorders and quality of life as a way to increase the quality of evidence and strength of the recommendation.

It is also important to highlight that in the included studies, three items were the least attended according to the customized checklist of Downs and Black for the risk of bias assessment. Items 3 and 9 refer to “reporting” and item 26 refers to “internal validity”. Therefore, we recommend that future studies better describe the sample inclusion and exclusion criteria, as well as justifying and reporting the proportions of follow-up losses in order to reduce the risk of bias in observational studies.

## Strengths and limitations

Strengths: 1) The originality of the theme, given that the COVID-19 pandemic generated a significant and immeasurable impact on the population worldwide; 2) The target population, given the high prevalence of chronic neurodegenerative and demyelinating diseases [5] such as PD, MS, and AD; 3) The outcomes, since the identification of neuropsychiatric disorders and sleep disorders allows the implementation of the best treatment strategies, reflecting on the better quality of life of these populations; and 4) The methodology, given that it followed PRISMA guidelines, recommendations of the Cochrane Collaboration, the quality of evidence and strength of the recommendation by the GRADE approach, the risk of bias of the included studies by the customized checklist of Downs and Black, and the protocol was registered in PROSPERO.

Limitations: 1) The stages of PD disability [65] were not defined as an inclusion criteria and there was no distinction between the main types of MS possibly due to the difficulties and challenges of remote research with these populations during the COVID-19 pandemic; 2) Only observational studies were included, which does not allow cause-effect relationships to be established between the COVID-19 pandemic and outcomes investigated; 3) Based on the GRADE approach, the systematic review and meta-analysis studies of observational studies began as low quality of evidence due to a higher risk of bias, hindering the generalization of our results [25]; and 4) The limitations of qualitative research, such as the accuracy of the information collected and subjectivity of the analyses due to the interpretations of the authors.

## Conclusion

In general, the COVID-19 pandemic negatively impacted in individuals with PD and MS. Individuals with PD and MS had significantly higher levels of depression and stress compared to healthy controls. Individuals with MS showed a lower quality of life compared to healthy controls. On the other hand, the COVID-19 pandemic did not seem to have significant repercussions on anxiety in individuals with PD and MS, and sleep quality in individuals with MS when compared to healthy controls. Further studies are needed to investigate the impact of the COVID-19 pandemic in individuals with AD and sleep disorders (e.g., insomnia). Future studies should prioritize the standardization of the scales for the assessment of neuropsychiatric disorders and quality of life in individuals with PD and MS to increase the quality of evidence and strength of the recommendation.

## Data Availability

The datasets used and/or analysed during the current study available from the corresponding author on reasonable request.

## Abbreviations

AD: Alzheimer's disease
BAI: Beck Anxiety Inventory
CI: Confidence intervals
COVID-19: Coronavirus disease 2019
DASS: Depression, Anxiety, and Stress Scale
GAD: Generalized Anxiety Disorder
GRADE: Grading of Recommendations Assessment, Development, and Evaluation
HADS: Hospital Anxiety and Depression Scale
MS: Multiple Sclerosis
PD: Parkinson's disease
PHQ: Patient Health Questionnaire
PICO: Patient, Intervention, Comparison, and Outcome
PSS: Perceived Stress Scale
PRISMA: Preferred Reporting Items for Systematic Reviews and Meta Analyses
PROSPERO: Prospective Register of Systematic Reviews
SARS-CoV-2: Severe acute respiratory syndrome coronavirus 2
SMD: Standardized mean differences
